# Two-Photon Luminescence and Second Harmonic Generation from Gold Micro-Plates

**DOI:** 10.3390/s141018328

**Published:** 2014-09-29

**Authors:** Xu Wang, Hao Shi, Naiyin Wang, Lianghui Cheng, Ying Gao, Lu Huang, Yuqiang Jiang

**Affiliations:** 1 Institute of Genetics and Developmental Biology, Chinese Academy of Sciences, West Beichen Road NO.1, Chaoyang District, Beijing 100101, China; E-Mails: wangxuscnu@genetics.ac.cn (X.W.); huanglu@genetics.ac.cn (L.H.); 2 Institute of Optoelectronic Materials and Technology, South China Normal University, West Zhongshan Avenue NO.55, Tianhe District, Guangzhou 510631, China; E-Mails: shihao159@sina.com (H.S.); naiyin.wang@gmail.com (N.W.); chenglhhui@163.com (L.C.); y.gao@genetics.ac.cn (Y.G.)

**Keywords:** gold micro-plates, two-photon luminescence, second harmonic generation

## Abstract

Micron-sized gold plates were prepared by reducing chloroauric acid with lemongrass extract. Their two-photon luminescence (TPL) and second harmonic generation (SHG) were investigated. The results show that the TPL and SHG intensity of gold plates is dependent on the wavelength and polarization of excitation laser. The TPL intensity of gold plates decreases with the increase of the excitation wavelength except for a small peak around 820–840 nm, while SHG intensity increases with the excitation wavelength redshift. In addition, it is found that the TPL intensity of the gold plate’s edge is related with the angle between the edge orientation and the polarization direction of the excitation light. The TPL intensity increases with the angle increase from 0° to 90°.

## 1. Introduction

Metal nanoparticles have been extensively applied in the bio-imaging, photothermal therapy, photonic devices and solar cells due to their plasmon-modulated optical properties [1–6]. The TPL and SHG intensity from low-symmetry metal nanoparticles depend greatly on the excitation wavelength and polarization [6–9], and can be drastically enhanced by the localized surface plasmon resonance (LSPR) [10,11]. Surface Plasmon Resonance imaging (SPRi) is an established tool in the life sciences. It offers a new generation of label-free bio-molecular analysis, providing information on the dynamic processes (association and dissociation), binding affinity, analyte concentration and real-time molecule detection. As typical sensors, the properties of the noble metal film and nanoparticle arrays have been well investigated and applied in the SPRi [12–16], while the noble metal micro-structures need further studies.

Micron-sized metal structures (MMS) have been applied in some fields [17–20]. MMS possesses prominent advantages for instance their SPR are obvious and can be resolved by an optical microscope. In this paper, we investigated the TPL and SHG of micron-sized gold plates excited by a wavelength tunable femtosecond laser with a two-photon fluorescence microscope, and found the dependence of the TPL and SHG intensity on wavelength, power and polarization of the excitation light.

## 2. Experimental Methods

A few methods have been proposed to prepare gold nanoparticles [21–23]. The gold micro-plates used in our experiment were prepared by reducing chloroauric acid with lemongrass extract. An amount of 15 g of thoroughly washed lemongrass leaf was boiled in 300 mL deionized water for 10 min to get 150 mL lemongrass extract. An amount of 2 mL filtrated lemongrass extract (the aperture of filter membrane is 0.47 μm) was added into 45 mL aqueous HAuCl_4_ solution with a concentration of 1 mM, and then the total volume was set to 65 mL with deionized water. The mixture was standing for 12 h at room temperature.

**Figure 1.**
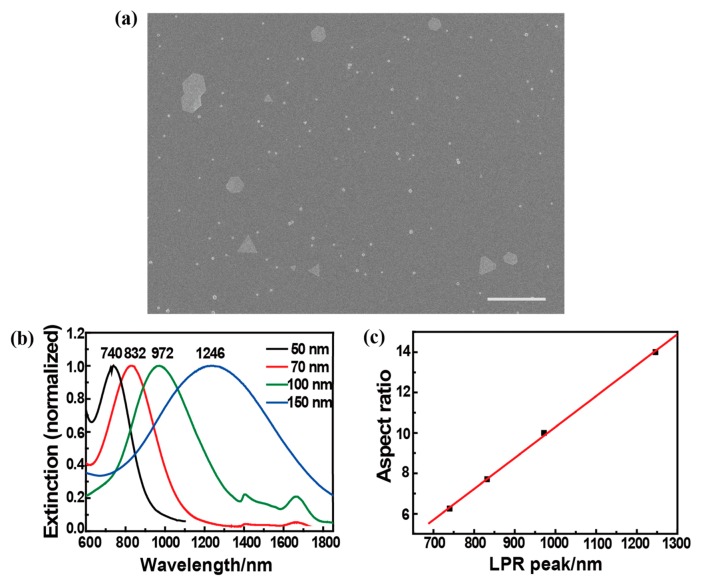
(**a**) The gold micro-plates prepared by the lemongrass extract reducing chloroauric acid. Scale bar: 10 μm; (**b**) The extinction spectra of various sized triangular gold plates, and the color of curve indicates the different base-length of gold plates; (**c**) The dependence of aspect ratio (base length to thickness of the plate) on the SPR wavelength.

As shown in Figure 1, the product contains some micron-sized gold plates and small gold particles. The base length of gold micro-plates varies from 1 to 3 μm. We have measured the extinction spectra of triangular gold nanoplates, as shown in Figure 1b,c. The peak value related to the SPR wavelength redshifts greatly with the increase of base length of nanoplate. Based on these results, the SPR wavelength of the micron-sized gold plates is deduced to be located at 5 μm or longer, which is far away from the excitation wavelength of a femtosecond laser.

**Figure 2.**
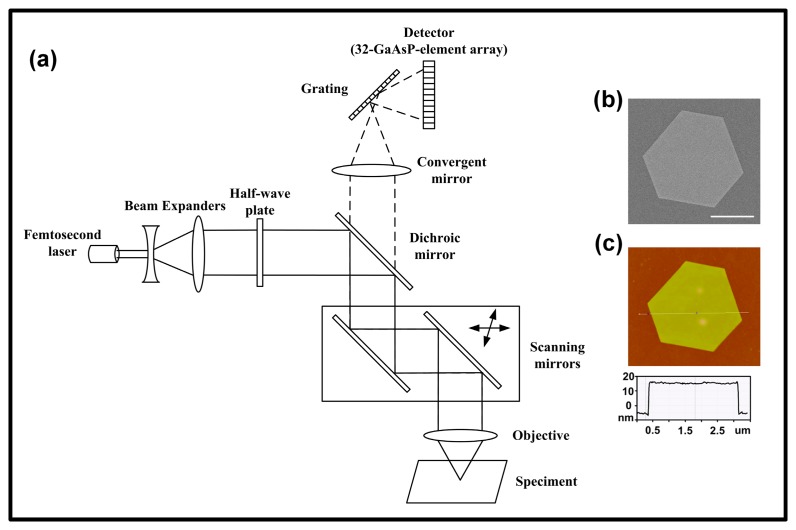
(**a**) The schematic diagram of the experimental setup, a two-photon fluorescence microscope. Solid lines indicate the excitation light, while dashed lines show the path of the emission signal from the sample; (**b**) and (**c**) The SEM and AFM images of a gold plate. The thickness of the gold plate is about 20 nm. Scale bar: 1 μm.

A two-photon fluorescence microscope (Zeiss LSM 780 NLO) was employed to measure the TPL and SHG from the gold micro-plates. The schematic diagram of the setup is shown in Figure 2a. The pulse width of a femtosecond laser is about 100 fs and its repetition rate is 80 MHz. The wavelength is tunable from 690 to 1040 nm. Excitation power can be selected by adjusting the femtosecond laser transmittance from 0% to 100%. Emission spectra can be detected by a 32-GaAsP-element-array detector built in the microscope. The spectral range of the detector was from 400 nm to 690 nm, and the resolution was 9 nm. A half-wave plate was used to change the linear polarization direction of the femtosecond laser. The gold plates were stuck on surface of a coverslip. After measurements of their optical properties, the size of gold plate was determined by a scanning electronic microscope (SEM) and an atomic force microscope (AFM). Figure 2b,c shows the morphology and thickness of a gold plate, respectively.

## 3. Results and Discussion

**Figure 3.**
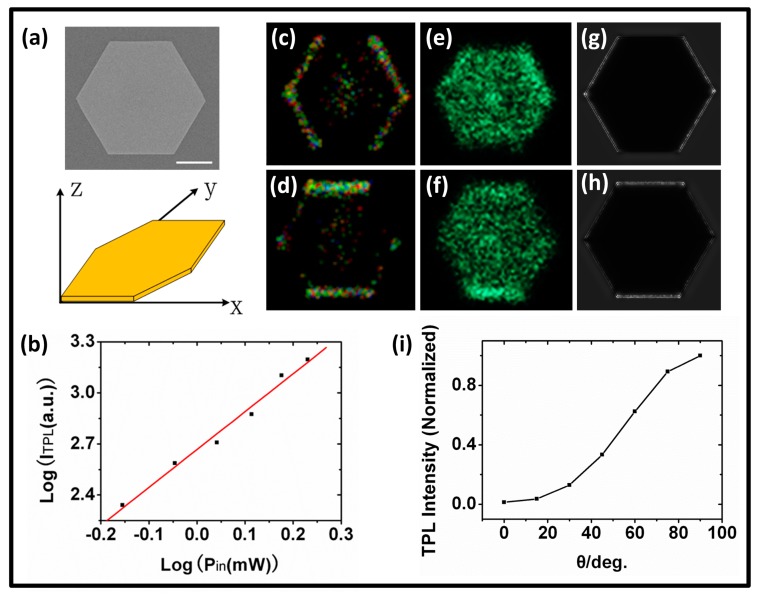
(**a**) The SEM image of a single gold plate, and its orientation in a 3-D coordinate system; (**b**) The logarithmic plot of the TPL intensity versus the excitation power. The dots are the measured data and the expression of the fitted curve is Log(I_TPL_) = 2.66 + 2.23 Log(P_in_); (**c**) and (**d**) TPL distributions of the gold plate at different excitation polarizations (parallel and perpendicular to the *x* axis); (**e**) and (**f**) The SHG image of the gold plate at different excitation polarizations (parallel and perpendicular to the *x* axis); (**g**) and (**h**) Simulated electric-field distributions of the gold plate at different excitation polarizations (parallel and perpendicular to *x* axis); (**i**) The fitted curve of the simulated TPL intensity against the angle θ between the edge orientation and the excitation polarization. The TPL intensity at different angles is normalized with respect to the intensity value at 90°. Scale bar: 1 μm.

Representatively, we studied the optical properties of a hexagonal gold plate (Figure 3a), experimentally and theoretically. The luminescence from the plate was measured by a 32-GaAsP-element array detector, thus, either the intensity or spectrum of luminescence can be determined. As shown in Figure 3b, the fluorescence intensity nonlinearly increases with increasing excitation power. Approximate quadratic dependence of the fluorescence intensity on the excitation power is observed, which proves that the observed luminescence is excited by the two-photon absorbance.

In the following, we investigated the dependence of TPL and SHG from the gold micro-plate on the polarization and wavelength.

### 3.1. Dependence of the TPL and SHG from Gold Micro-Plates on Excitation Polarization

For convenience, three-dimensional coordinates are defined, as shown in Figure 3a, the gold micro-plate is in the *x*-*y* plane, and the excitation light propagates along the negative *z*-axis direction. The polarization direction of the femtosecond laser on the sample was set at 0° or 90° with respect to *x* axis by adjusting the half-wave plate. Gold plates were excited with the excitation wavelength of 820-nm and the power of 1.5 mW after objective lens in the two polarization directions. Figure 3c–f shows the TPL and SHG images with the excitation light polarization at 0° or 90° relative to the *x* axis. It is found that the TPL distribution of the gold plate is dependent on the excitation polarization, and the TPL intensity on edges of the gold plate is dependent on θ, the angle between the edge orientation and the polarization direction of the excitation light. The upper edge of the hexagonal gold plate, for example, emits the weakest TPL at θ = 0°, while the strongest at θ = 90°. Considering the TPL emission mechanism of gold particles [11], the spatial-distribution property of the TPL of the gold micro-plate should be related to its local electric field enhancement.

We calculated the electric-field distributions of the gold plate excited by the plane wave (electric intensity 1 V/m). The excitation wavelength is also set to be 820 nm. The partial differential equations for the steady-state distribution were solved using the finite element method. The refractive index used for Au was that reported by Palik [24]. Figure 3g,h shows the distribution of the electric field obtained with the excitation light polarized at 0° or 90° relative to the *x* axis, which is well consistent with the experimental results.

The electric field distributions at various polarization directions (0°, 15°, 30°, 45°, 60°, 75°, and 90° with respect to the *x* axis) were calculated. As the TPL intensity is proportional to the fourth power of the electric field, the dependence of the TPL intensity at the upper edge of the gold plate against the angle θ can be calculated (Figure 3i). The simulated result shows that the TPL intensity increases with the angle from 0° to 90°. The results verify that spatial-distribution properties of TPL on the edges of gold plates are attributed to the local electric field enhancement.

The excitation-polarization dependence of the electric-field enhancement at the edge of the gold micro-plate is similar to scratched gold films, which have already been investigated [25,26]. When the polarization direction of the excitation light is perpendicular to the upper-edge orientation, surface plasma may oscillate between the upper and lower edges and the electric-field enhancement will occur on the edges. When the polarization direction of the excitation light is parallel to the upper-edge orientation, the surface plasmon oscillation between the upper and lower edges is not expected and the electric field at these edges cannot be enhanced.

**Figure 4.**
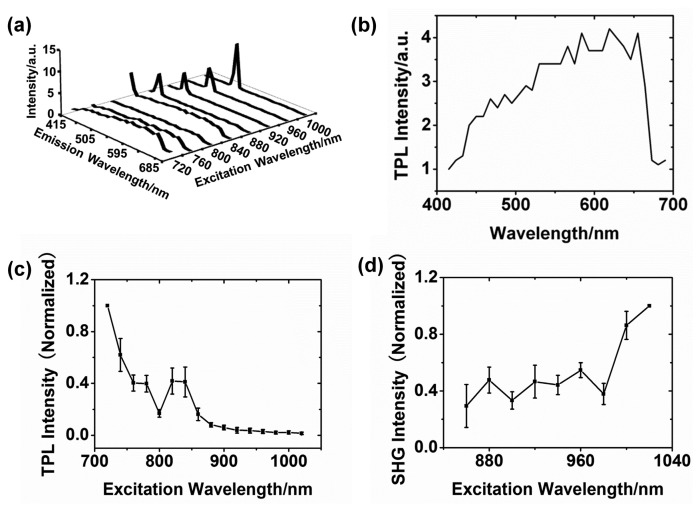
(**a**) The emission spectra of the gold plate at the different excitation wavelengths and same polarization (perpendicular to the *x* axis); (**b**) The TPL spectrum of the gold plate at the 720-nm excitation wavelength; (**c**) The TPL intensities at 600 nm versus the excitation wavelength. The TPL intensity is normalized with respect to the intensity value at the 720-nm excitation wavelength; (**d**) The SHG intensities *vs.* the excitation wavelength. The SHG intensity is normalized with respect to the intensity value at the 1020-nm excitation wavelength.

SHG images are shown in Figure 3e,f. In contrast to TPL, the SHG signal is much stronger and can be observed on the whole upper surface. This can be verified by the spectral measurement, as shown in Figure 4a. The SHG on some edges is more obvious, that also coincided with the distribution of electric field.

### 3.2. Dependence of the TPL and SHG from Gold Micro-Plates on Excitation Wavelength

Gold plates were excited at different excitation wavelengths (720–1020 nm, interval 20 nm) with the 1.5-mW power, and emission spectra were detected by the 32-GaAsP-element-array detector built in the microscope. Partial spectra of a gold plate are shown in Figure 4a. When an excitation wavelength is larger than 800 nm, SHG signal can be observed in the spectra. The sharp peaks in these curves result from SHG signals. As we mentioned above, the SHG is much stronger than TPL. Figure 4b shows one spectrum of the gold plate at the 720-nm excitation wavelength. Its characteristics are quite consistent with the previous studies [11]. Luminescence emission of the gold plate is dependent on the excitation wavelength. The TPL intensity tends to decrease with the excitation wavelength redshift, but interestingly, there is a small peak around the 820–840 nm, as shown in Figure 4c.

In addition, we also measured the SHG intensity over the excitation wavelength range 860–1020 nm, and the results are shown in Figure 4d. The SHG intensity of gold plates exhibits an increasing tendency with the redshift of the excitation wavelength. The SHG signal extensively exists on the whole upper surface of the gold micro-plate, while enhancement tends to occur at some edges.

The similar results were obtained from various gold micro-plates of the different size (edge length 1–3 μm) and shape (triangular or hexagonal). The error bars in Figure 4c,d indicate the variation of luminescence intensities caused by the difference of size and shape. Despite some minor diversity, the tendencies of the curves from different micro-plates are identical.

As the size of gold micro-plates used in the experiments is relative large, the excitation-wavelength dependence of the SHG signal from the gold plates may be explained with the SHG theory refer to a gold film. According to Krause [27], the SHG tensor element of the gold film is:

(1) $$\chi^{2}\approx \frac{e}{16\pi m\omega^{2}}(\varepsilon_{\omega }-1)$$

where *e* is the absolute value of the charge of a single electron, and *m* is the electronic mass. It can be found that the magnitude of the surface χ^2^ element is excitation-frequency dependent. The relation of dielectric *ε_ω_* with the frequency is as follows [28]:

(2) $$\varepsilon_{\omega }=1-\frac{{\omega_{P}}^{2}}{\omega^{2}+i\frac{\omega }{\tau }}$$

where *ω_p_* is the plasmon frequency, and *τ* is the conduction-electron relaxation time. The SHG intensity *I*_2_*_ω_* can be expressed as [29]:

(3) $$I_{2\omega }\propto {I_{\omega }}^{2}{\left|\chi^{\left(2\right)}\right|}^{2}$$

where *I_ω_* is the intensity of the fundamental excitation light. We can get the frequency-dependent expression of *I*_2_*_ω_* by combining Equations (1)–(3).

(4) $$I_{2\omega }\propto {I_{\omega }}^{2}\frac{e^{2}{\omega_{p}}^{4}}{256\pi^{2}m^{2}\omega^{6}(\omega^{2}+\frac{1}{\tau^{2}})}$$

Thus, the SHG intensity increases with the decrease of the excitation frequency, which is qualitatively consistent with our experimental results.

## 4. Conclusions

We have investigated the TPL and SHG properties of micron-sized gold plates. The TPL and SHG intensities were found dependent on the polarization direction and wavelength of the excitation light, and the experimental results are consistent with the simulation ones. The TPL intensity on the edge of gold plate increases with the angle between edge and polarization direction. These results, not only broaden our understanding of micron-sized gold plates, but also provide some potential applications. For example, the gold micro-plates can be used to control the molecular fluorescence emission, and the gold micro-plates array can be used as a sensor for the detection of molecule concentration.
